# Views and Uses of Sepsis Digital Alerts in National Health Service Trusts in England: Qualitative Study With Health Care Professionals

**DOI:** 10.2196/56949

**Published:** 2024-10-15

**Authors:** Runa Lazzarino, Aleksandra J Borek, Kate Honeyford, John Welch, Andrew J Brent, Anne Kinderlerer, Graham Cooke, Shashank Patil, Anthony Gordon, Ben Glampson, Philippa Goodman, Peter Ghazal, Ron Daniels, Céire E Costelloe, Sarah Tonkin-Crine

**Affiliations:** 1 Nuffield Department of Primary Care Health Sciences Medical Division University of Oxford Oxford United Kingdom; 2 National Institute for Health and Care Research (NIHR) Health Protection Research Unit in Healthcare Associated Infections and Antimicrobial Resistance University of Oxford Oxford United Kingdom; 3 Team Health Informatics Institute of Cancer Research London United Kingdom; 4 University College Hospital London United Kingdom; 5 Oxford University Hospitals NHS Foundation Trust Oxford United Kingdom; 6 Imperial College Healthcare NHS Trust London United Kingdom; 7 Chelsea and Westminster Hospital London United Kingdom; 8 PPI representative Oxford United Kingdom; 9 School of Medicine Cardiff University Cardiff United Kingdom; 10 UK Sepsis Trust and Global Sepsis Alliance Birmingham United Kingdom

**Keywords:** digital alerts, electronic health records, computerized clinical decision support systems, sepsis, patient deterioration, decision-making, secondary care, emergency care, intensive care, England, qualitative study

## Abstract

**Background:**

Sepsis is a common cause of serious illness and death. Sepsis management remains challenging and suboptimal. To support rapid sepsis diagnosis and treatment, screening tools have been embedded into hospital digital systems to appear as digital alerts. The implementation of digital alerts to improve the management of sepsis and deterioration is a complex intervention that has to fit with team workflow and the views and practices of hospital staff. Despite the importance of human decision-making and behavior in optimal implementation, there are limited qualitative studies that explore the views and experiences of health care professionals regarding digital alerts as sepsis or deterioration computerized clinician decision support systems (CCDSSs).

**Objective:**

This study aims to explore the views and experiences of health care professionals on the use of sepsis or deterioration CCDSSs and to identify barriers and facilitators to their implementation and use in National Health Service (NHS) hospitals.

**Methods:**

We conducted a qualitative, multisite study with unstructured observations and semistructured interviews with health care professionals from emergency departments, outreach teams, and intensive or acute units in 3 NHS hospital trusts in England. Data from both interviews and observations were analyzed together inductively using thematic analysis.

**Results:**

A total of 22 health care professionals were interviewed, and 12 observation sessions were undertaken. A total of four themes regarding digital alerts were identified: (1) support decision-making as nested in electronic health records, but never substitute professionals’ knowledge and experience; (2) remind to take action according to the context, such as the hospital unit and the job role; (3) improve the alerts and their introduction, by making them more accessible, easy to use, not intrusive, more accurate, as well as integrated across the whole health care system; and (4) contextual factors affecting views and use of alerts in the NHS trusts. Digital alerts are more optimally used in general hospital units with a lower senior decision maker:patient ratio and by health care professionals with experience of a similar technology. Better use of the alerts was associated with quality improvement initiatives and continuous sepsis training. The trusts’ features, such as the presence of a 24/7 emergency outreach team, good technological resources, and staffing and teamwork, favored a more optimal use.

**Conclusions:**

Trust implementation of sepsis or deterioration CCDSSs requires support on multiple levels and at all phases of the intervention, starting from a prego-live analysis addressing organizational needs and readiness. Advancements toward minimally disruptive and smart digital alerts as sepsis or deterioration CCDSSs, which are more accurate and specific but at the same time scalable and accessible, require policy changes and investments in multidisciplinary research.

## Introduction

### Background

Sepsis is an uncontrollable response of the body to an infection, whereby the immune system starts attacking its own tissues and organs leading to organ dysfunction [1]. Sepsis is a common cause of serious illness and death. There are an estimated 918,000 hospital admissions with suspected sepsis, up to 250,000 cases of sepsis, and 48,500 deaths associated with sepsis in the United Kingdom each year [2]. Similarly, high levels of sepsis are reported internationally [3,4], with 11 million sepsis-related deaths calculated for 2017, representing approximately 20% of all deaths globally [5,6]. Sepsis is recognized by the World Health Organization as a global health priority [7].

Sepsis symptomatology can look like that of other conditions, potentially delaying and misleading diagnosis and treatment. As such, sepsis demands prompt intervention to avert poor outcomes [8]. Evidence has shown that timely, appropriately targeted, intravenous antibiotics are effective in improving outcomes for patients with sepsis [9-11]. On the basis of this evidence, hospitals in the United Kingdom have been set targets to rapidly diagnose and administer intravenous antibiotics [12,13]. Other countries have introduced national guidance for sepsis [14]; and the International Surviving Sepsis Campaign updated guidelines for the management of sepsis in 2021. These latter guidelines recommend the adoption of performance and quality improvement initiatives and of protocols and screening tools to improve and accelerate the identification and treatment of sepsis in hospitals [8,15], although these processes remain challenging and suboptimal [16,17].

### Prior Work

Interlocking with the complexity of sepsis and the clinical intervention required [1], factors linked to the professional profile and the work environment affect the timely management of sepsis in secondary care. Previous qualitative research with health care professionals (HCPs) has highlighted limits in professionals’ capacity to identify sepsis, difficulties in hand over of patients, and errors in communication [18-22]. Time pressures, the intense workload, and the complex clinical environment are all barriers which need to be overcome; simultaneously, well-coordinated multidisciplinary work and effective leadership are necessary to combine smoothly for effective sepsis management [2,18,20,21].

To support rapid sepsis diagnosis and treatment, screening tools have been proposed. These include the quick sepsis-related organ failure assessment [qSOFA; 1]; systemic inflammatory response syndrome (SIRS) criteria [23,24]; and in the United Kingdom, the national early warning score (NEWS) in 2012 [25,26], updated in 2017 as NEWS2 [12,27]. More recently, machine learning algorithms for early recognition of sepsis have been developed, performing better when compared with the aforementioned sepsis scoring tools [28,29].

With the expansion of electronic health records (EHRs) across the UK National Health Service (NHS), screening tools for sepsis have been embedded into hospital digital systems (DSs) as digital alerts. These digital tools are also known as computerized clinical decision support systems (CCDSSs) and are meant to enhance clinical decision with both targeted and general information [30], but there are also considerable concerns about alert fatigue [31]. There are different ways in which digital alerts, including sepsis or deterioration CCDSSs, interface with users. A total of 3 categories have been identified [32]. Hard-stop intrusive alerts pop up and either prevent users from taking any action or allow them to proceed only with the override of a third party, such as a senior decision maker. Soft-stop intrusive alerts allow the user to proceed after entering a response, often from a multiple-choice box. Nonintrusive, passive alerts do not pop up or interrupt the user workflow or require any interaction on the part of the user. Sometimes these are in-line, for example, an alert appearing in a row of an electronic patient list.

Previous evidence on the effect of digital alerts as sepsis or deterioration CCDSSs (henceforth, sepsis digital alert) on care quality and patient outcomes offered mixed results [33]. Work in NHS trusts in England demonstrated that the introduction of digital sepsis screening tools with their accompanying alerts was associated with an increase in timely treatment and reduction in risk of mortality in 2 trusts [34,35]. Some studies, mainly conducted in the United States, have demonstrated an increase in patients receiving timely intravenous antibiotics [36,37] and a decrease in in-hospital mortality [38-40] and length of stay [36,41,42], but others have not found any impact either on mortality [37,41,43,44] or hospital length of stay [39].

Sepsis digital alerts, which are embedded into the hospital EHRs DS, fit into the highly complex workflow of the management of sepsis, as any other CCDSSs. Qualitative literature exploring HCPs’ views and experiences with sepsis digital alerts is scarce. A recent study [45] found that HCPs’ perceptions of a hypothetical sepsis digital alert are linked to the level of trust professionals had in the alert; HCPs’ trust was enhanced both by their previous experience with similar alerts and by being engaged in implementation and training initiatives about the sepsis digital alert. In another US-based study, physicians in a pediatric emergency department (ED) were interviewed about reasons for accepting or rejecting a sepsis electronic best practice alert: two-thirds of participants considered nonpatient factors relevant specific to the ED environment, individualized practice patterns, the digital tool design, and education [46].

### Goal of This Study

The emerging evidence of the contribution of sepsis digital alerts is promising but still limited; there are no validated digital tools available to NHS trusts, with scarce evidence as to which tool to use and their effect on patient outcomes. In addition, the introduction of sepsis digital alerts is often accompanied by treatment plans, under the aegis of antimicrobial stewardship, and by implementation and quality improvement initiatives and education and training for HCPs; while these have been shown to yield positive results [34,47,48], little is known on how these actions affect the implementation and impact of the alerts.

This study aimed to explore the views and experiences of HCPs on the use of sepsis digital alerts and to identify barriers and facilitators to the implementation and use of sepsis digital alerts in NHS hospitals.

## Methods

### Study Design

This is a qualitative, multisite study with semistructured interviews with HCPs and unstructured observations of HCPs working in hospitals. It is nested within a broader program of research seeking to evaluate the impact of sepsis digital alerts on patient outcomes and staff activity in NHS hospitals, the Digital Alerting for Sepsis (DiAlS) study.

### Sample and Settings

A combination of purposive and convenience sampling was used. A total of 3 NHS trusts were selected as sites for this qualitative study from the 6 hospitals involved in the DiAlS study. The 3 sites were chosen with consideration of diversity in the EHRs DS and in the sepsis digital alerts, previous evidence on the evaluation of sepsis digital alerts [34], and implementation and quality improvement initiatives. Trusts’ coinvestigators were asked to identify and invite potential participants for interviews from their trusts. Participants were sampled to include a variety of job roles and a range of hospital units (EDs, outreach emergency response teams, and acute and intensive care units [ICUs]). In 2 sites (1 and 2), observations were conducted, and coinvestigators supported the research team to this end providing initial liaising with relevant colleagues in ED and the outreach team.

### Data Collection

All data were collected between November 2022 and July 2023 by RL and AJ Borek. Most semistructured interviews with HCPs were conducted remotely using videoconferencing software (Microsoft Teams); the remaining were conducted in person at the participant’s place of work. The topic guide was developed from the study objectives with input from the wider research team and the study patient and public involvement representative (Multimedia Appendix 1). Questions asked HCPs about their experiences of identifying and managing patients with sepsis and about their views and experiences of using sepsis digital alerts. Where relevant, HCPs were asked about their experience of developing and implementing sepsis digital alerts. Interviews lasted between 30 and 75 (mean 52) minutes, were audio recorded, and were professionally transcribed verbatim and pseudonymized.

Unstructured observations [49] sought to observe clinical practice to see how sepsis digital alerts fitted into the workflows of HCPs in different roles. We sought to assess what impact they had on clinical decision-making and to identify whether sepsis digital alerts were used differently by HCPs in different roles. A total of two types of observation were done: (1) observations of practice in EDs, with occasional informal conversations, and (2) one-to-one shadowing of an HCP in outreach teams, with more frequent dialogue. Either the HCP who expressed interest in being shadowed or the ED head or manager consented in writing before the observations. The observations lasted between 2 and 4 hours. Paper-and-pencil notes taken during the observations were anonymized and typed up.

### Data Analysis

Data analysis began concurrently during data collection and was supported by NVivo (version 12; QSR International). Data from both interviews and observations were analyzed together inductively using thematic analysis [50,51]. Similarities and differences between transcripts were assessed using a constant comparison approach [52]. A codebook was developed to code the whole data set, across the 3 sites. Codes were compared with one another to create categories, grouping similar codes together. All categories were clearly named to ensure that only related data were included in that category. However, some codes and categories remained site specific to allow for comparisons between sites. Regular core team (RL, AJ Borek, and STC) meetings accompanied data collection and analysis phases to deliberate on the codebook and the data analysis and to follow an iterative approach, which is documented in a number of Microsoft Word documents with annotations and comments for auditing purposes. We referred to the 8 quality criteria for qualitative research to ensure rigor and trustworthiness of our process [53].

### Ethical Considerations

The wider program of research, the DiAlS study, of which this study is a part, was reviewed and approved by the UK NHS Health Research Authority (Project ID—288,328). This qualitative work stream was further separately reviewed and approved by the Research Governance, Ethics and Assurance Team of the University of Oxford and the UK NHS Health Research Authority of England and Wales (Project ID 313699-22/PR/1020). The research teams of each trust reviewed and approved the study at the site level. Full verbal or written consent was obtained from all participants included in this study. Data were pseudonymized. Participants were offered a voucher to thank them for their time. The monetary value of the voucher was £20; US $25.

## Results

### Overview

We interviewed 22 HCPs: 8 (36%) from site 1, and 7 (32%) from each of the other 2 sites (Table 1). In site 1 and 2, a total of 12 observation sessions were also undertaken: 5 (42%) in the EDs and 7 (58%) sessions involved shadowing professionals, 3 (25%) of whom completed an interview. We identified 3 themes about HCPs’ views and use of their sepsis digital alerts and a fourth theme capturing the complexity of how the hospital environment affected sepsis digital alerts’ use across the 3 sites. The first 3 themes feed into the fourth theme, which allows a comparison between the 3 NHS trusts involved in this study. The presentation of the results was structured in this way as each section is seen as building on the previous one and supporting the following one.

**Table 1:** Characteristics of interview participants (N=22).

Site	Participants, n (%)	Declared gender (women), n (%)	Age (y), mean (range)	Job title and hospital unit (n)	Years of experience, mean (range)	Years of use of digital alerts, mean (range)
Site 1	8 (36)	3 (14)	43.5 (34-55)	Nurses and OT^a^: n=3 Nurse and ED^b^: n=1 Consultants and ICU^c^: n=3 Consultant and ED: n=1	10 (7-18)	6 (4-10)
Site 2	7 (32)	5 (23)	36 (29-42)	Nurse and OT: n=2 Nurse and ED: n=1 Nurse and AMU^d^: n=1 Consultant and OT: n=1 Consultant and ED: n=1 Consultant and ICU n=1	6 (3-8)	5 (1-7)
Site 3	7 (32)	7 (32)	43 (30-54)	Nurses and OT: n=3 Nurse and ED: n=1 Nurse and AMU: n=1 Consultants and ED: n=2	9 (1-20)	7 (5-10)
Total	22 (100)	15 (68)	41 (29-55)	Nurses: n=13 Consultants: n=9 OT: n=9 ED: n=7 AMU: n=2 ICU: n=4	11 (1-20)	6 (1-10)

^a^OT: outreach team.

^b^ED: emergency department.

^c^ICU: intensive care unit.

^d^AMU: acute medical unit.

### Theme 1: Alerts Nested Within EHRs Support Decision-Making

All participants liked that sepsis digital alerts were nested within the DS of the EHRs shared across a trust. Having patient data all in 1 place, including clinical history, patients’ trends during their hospital stay, preconditions, comorbidities, test results, and various digital alerts or scores, was described as useful to more quickly and safely build a picture of the patients who are flagged by the sepsis digital alert. This was regarded as enabling better decision-making and quality care:

We’ve been electronic in ICU for a long time but, of course, the interaction has been difficult because of the wards being paper-based and now having everything available everywhere is incredibly helpful. So, the cutting out of searching for information, it makes things much more efficient and I think as a result, much safer.

ICU consultant, site 1

All participants underlined that the sepsis digital alert in the patients’ EHRs were supporting, and by no means substituting, clinical decision-makers, whose knowledge and experience were of paramount importance in sepsis identification and management. Sepsis digital alerts were seen as “a piece of the puzzle” (outreach team nurse, site 3), neither intended for, nor leading to, the formulation of a diagnosis or clinical decision in isolation:

I think it’s another objective piece of the puzzle that will support junior members to ask for more help because they cannot ignore it, which is helpful I think, when they are wondering how to identify that someone is unwell.

Outreach team nurse, site 3

I will go and look in the obs chart for the patient to figure out what obs would have triggered the alerts and if there’s congruence. So, does the presentation and the obs match the sepsis digital alert?...The more senior you get the more nuance you’re looking for.

ED consultant, site 3

Some participants placed greater emphasis on the teamwork support function of the sepsis digital alert. They valued that they could access information related to clinical observations and the actions of their colleagues, reach out to them if needed, and factor these data into their own decisions. Other participants emphasized the monitoring support function of the sepsis digital alert; having a quick synopsis of the condition of all the patients in a hospital unit allowed better patient prioritization and management of workload:

Nowadays, I am not looking at six patients, which I can go you, you, you, you, you. I’m looking at 60 and how do I look at those patients? I can’t. How do I look at them? I look at them electronically and you know, the digital system and the alert enabled me, despite not flawlessly, to build a really good impression about what the acuity is and where the danger is within my department.

ED consultant, site 1

At the operational level, participants described how sepsis digital alerts are embedded within a complex, multimodal way of working, which includes bleepers, mobile phones, and landline phones for communications between hospital units’ team and with the laboratories; paper notes in absence of available computers to quickly annotate patients’ observations before uploading them onto the DS; and face-to-face interactions, including for very urgent escalations:

The triage nurse is quite far from us...So, if they’re very worried and they can’t get through on our phone, then they come and find us in person and walk to us and ask for advice. Usually, because they’re also quite busy, they usually try to ring us and just make us aware of the patient on the screen and they just say, please can you prescribe paracetamol, antibiotics, and request patient review. Then I can do it over the phone. In A&E, we don’t really hold bleeps. Bleeps are more for the ward team.

ED consultant, site 3

### Theme 2: Alerts Are Reminders That Lead to Context-Dependent Actions

Participants viewed sepsis digital alerts as useful to remind and prompt HCPs of a number of actions, from reviewing a patient’s information in the EHRs to visiting them in person. In general, participants reported that sepsis digital alerts’ utility decreased with increasing staff training and experience with sepsis cases, and with the higher senior HCP:patient ratio of certain units, such as acute or intensive care. More junior HCPs said that sepsis digital alerts afforded them with greater confidence to further investigate and interpret why sepsis digital alerts triggered, which could lead them to follow the sepsis protocol and to escalate to senior decision-makers:

Thinking back [to] being a junior, one of the big lessons is learning to recognise a critically sick patient, and that takes experience, so having some hard parameters to hang your hat on is really helpful, because I think we all remember you know running to get help when actually things were fine.

ICU consultant, site 1

For senior participants who “have a greater cognitive load” (acute medical unit nurse, site 3), the sepsis digital alerts were described as reminders to avoid missing actions for patients in their department:

I think the alert itself is really helpful, I think particularly for when you’re looking through a patient list, the whiteboard of patients, to have the visual prompt there of somebody that may be way down the list in view of time to be seen, waiting to be seen, but if they’ve got that alert on the system then generally a senior registrar will pick those patients out and review their case and potentially start the right treatment before the patient is fully assessed, so I think the prompt certainly helps with that identification.

AMU nurse, site 3

HCPs’ specialty and experience, and hospital units hosting different patient cohorts—with varying conditions, treatments, and lengths of stay—were seen to play a role in the use and views of sepsis digital alerts. Thus, some participants commented that they found sepsis digital alerts unspecific and oversensitive, which potentially could lead to overtriaging, overreferring, and, more rarely, overtreating patients:

In a major trauma ward the alert could be triggering because, I don’t know, they’ve got local pressure...It doesn’t necessarily mean they’ve got sepsis, it’s very injury-related. Those nurses might find it a little bit frustrating because they will be like, I don’t need to go and give antibiotics straight away, with the sepsis six treatment, because it is irrelevant for this quota of patients.

AMU nurse, site 3

Nevertheless, participants highlighted that this also had the positive implication of increasing inter- and intrateam communication, and reducing the risk of missing patients:

The threshold for which EHR alerts are generated is very, very low so there’s different criteria that could match together to generate that alert and, therefore, we do get a degree of inappropriate patient alerts and referrals. But equally it’s much better to because if we screen those patients, it’s better that we have more rather than missing some...with less sensitive criteria.

Outreach team nurse, site 2

### Theme 3: Improving Alerts and Their Introduction

Most participants expressed the importance of sepsis digital alerts being easy to use and accessible; some participants underlined that a user-friendly sepsis digital alert was important so that new staff could be trained more easily and quickly in its correct use. Other participants felt that ergonomic sepsis digital alerts were more likely to facilitate HCPs’ work and teamwork and to be acknowledged. Conversely, complicated interfaces and pop-up sepsis digital alerts were seen to interrupt HCPs’ work, requiring several steps and with the risk of confusing, desensitizing, and irritating users. In result, sepsis digital alerts were reported to be overridden and ignored. By contrast, some participants warned against the alerts being used in excess and deskilling hands-on practice because HCPs, especially those more junior, may “become so fixated on the number that they forget the core part of some of their nursing skills” (outreach team nurse, site 1).

Thinking about potential improvements of sepsis digital alerts, participants suggested adding a checklist of what a colleague has or has not done, or key pieces of information regarding the patient that could be sent in an SMS text message when referring a patient. Participants also suggested quick training that could be accessed via a smartphone:

Currently to learn about sepsis, it requires you to log off the computer, to watch a video, to sit in front of a computer that you can only use with a plug, then it’s one of the barriers, but if you have a QR code, that anyone can access—because everyone got a smartphone at the moment—with a quick reference guide to what you need to do, but also some information about sepsis and also some training videos, what you’ve got to do, that will really help.

AMU nurse, site 3

In this respect, several participants, especially from the outreach teams, envisioned sepsis digital alerts embedded into portable devices so that HCPs could be reached when they are on the move. Other features of more advanced sepsis digital alerts that participants suggested included alerts being adjusted to the patient, factoring in their baseline parameters, comorbidities, and previous conditions or addressing relevant HCPs team; and alerts processing more information regarding the patient, transparently showing why they triggered and allowing for greater interaction with users:

What needs to happen is that when results come back and they’re horribly abnormal, the doctor or the nurse looking after the patient is alerted to that fact without them having to log into a computer...without having to remember the patient’s name and hospital number and then clicking through a load of tabs to find what the blood test is. Wouldn’t it be nice if there was some way that a person could be alerted directly that this particular patient has a particular problem?

ICU consultant, site 1

Many participants wished to have a sepsis digital alert embedded in a DS shared beyond the trust and across the community, ambulance service, and primary care. Participants felt that a widely integrated sepsis digital alert would reduce ED waiting, triaging, and handover times:

If there was a system whereby we could link up our different services and have those observations pulled through so that those patients that don’t get the alert when they have a set of normal obs in hospital, still have some other way of being flagged on the system that actually this concerning presentation was the case an hour ago in the community.

AMU senior nurse, site 2

Finally, participants found the implementation optimization and quality improvement initiatives around the sepsis digital alert useful and emphasized the need for ongoing training and education on sepsis identification and management. In all 3 trusts, at piloting and rollout, the sepsis digital alerts were iteratively adjusted based on staff feedback and regular training around sepsis. However, as some participants highlighted, several other “human factors” should be targeted by broader training aiming to change the organizational culture:

The predominant obstacle’s definitely human factors so where you get cultural norms within a ward and certain clinical areas...there’s just that kind of like, “Oh, we take care of our patients really well and they would never get sepsis” kind of attitude. There are clinical areas that we go to and there seems to be like a resistance [to] intervention from a specialist team because it’s like, “Oh, well, if it was that, then we would have noticed”...it’s that kind of assumption that they would know if the patient was going to deteriorate, which actually when you look at the evidence is not the case.

Outreach team nurse, site 2

### Theme 4: Contextual Factors Affecting Views and Uses of Alerts in the Trusts

This fourth theme brings together and present the first 3 themes against 4 sets of factors that affect the views and uses of sepsis digital alerts. Some of these factors differently combine in the context of each of the 3 trusts, allowing comparative reflections. The following quote encapsulates several of these factors constituting the complexity of the hospital environment:

It’s all human factor stuff. So, yes, we’ve got the digital alert, yes people know it might be sepsis, but what gets in the way is people. The context in which they’re working, their environment, the business of the ward, the acute conditions of the other patients, the demands on them. It swings both ways, if people are really not busy, not that that ever happens, but then people tend to do less. Then when they’re really, really busy things also get missed and they get a bit swamped.

Outreach team nurse, site 1

The first set is that of the factors pertaining to individual HCPs. As reported in theme 2, HCPs’ seniority and clinical specialty affect the use of sepsis digital alerts. On occasion, personal circumstances (eg, childcare duties and other nonprofessional commitments) can play a role in their decision-making.

The second set of factors relate to the hospital unit or department; these will have HCPs with specific training and specialty caring for certain types of patients, and this influences the use of sepsis digital alerts in these patient populations. For example, in intensive units, such as ED resuscitation and ICU, sepsis cases are seen more frequently, and more senior staff look after more severely ill patients so observations are taken more frequently. Participants reported that these aspects make the sepsis digital alert less relevant.

The third set is that of the factors pertaining to the trust. The workload:staffing ratio, along with the presence of senior decision-makers per patient, are specific to the hospital unit but also dependent on the overall management and resources of the trust. Several participants, from sites 2 and 3, raised the issue of delayed actioning of the sepsis digital alert due to heavy workload, and to the retrospective uploading of patients’ observations or clinical actions performed, resulting in lower performance toward meeting targets (eg, antibiotics within 1 h) at time of auditing:

The nursing notes said “IV access obtained. Antibiotics given” and then an hour or so later you see at 13:30pm antibiotic prescribed. 13:31pm antibiotic given. It all just seems a little bit like that’s all been done after the fact. So sometimes you just have to infer that it sounds like they were really on top of this, and they just left the documentation, rightly, till the end, but we can usually tell, and you can see that gap between prescription and administration, that’s often the bit that tips it over the 60 minutes.

Outreach team consultant, site 2

As highlighted in theme 3, the trust plays a role by investing in staff education or training about sepsis per se and sepsis digital alerts, in the implementation of quality improvement and in technological equipment, spanning from the number of computers available to introducing useful and accessible software programs.

The fourth set of factors is that of the digital tool itself, its features, and functionalities, which correlate with optimal use of sepsis digital alerts, as theme 3 encapsulated. Some aspects inherent to the sepsis digital alert are closely related to other factors, such as how ergonomic and accessible an alert is, which are linked to both trust and individual factors.

The 4 sets of factors differently combine in each of the trusts included in this study. Reading these in conjunction with the characteristics of each trust (Table 2) allows some comparative results, to which the unstructured observations have proved particularly enlightening.

In site 1, the DS was more recently introduced, with ED as the leading unit in the implementation; it is a straightforward system with hardly any intrusive alerts, but with a number of linkable phone apps and functions, such as the DS chat. The sepsis digital alert is a patient deterioration, nonsepsis specific, and passive alert. A nurse-led outreach team operates 24/7. The combination of these elements results in a perceived easiness-to-use of the DS and usefulness of the deterioration digital alert. Results indicate that the deterioration digital alert is acknowledged by HCPs and ignites the intended behaviors (eg, review, escalate, investigate, and visit). This positive pattern appears to occur even in the apparent absence of a major focus from the Trust on sepsis per se. Of note is that these same elements are related to, for some participants in site 1, an excessive reliance and use of the deterioration digital alert as a patient referral trigger; this means that the deterioration digital alert has been perceived to raise the number of patients’ referrals, sometimes unnecessarily.

In site 2, the sepsis digital alert is nested within a DS considered “clunky” (ED consultant, site 2) by some participants; some professionals expressed the opinion that digital alerts were too numerous and could cause them an “alert fatigue” (outreach team nurse, ED nurse, ICU consultant, and acute medical unit nurse). Sepsis has been a priority on the trust’s agenda, including via the development of an ad hoc algorithm for the sepsis digital alert, the establishment of an outreach team dedicated to sepsis, and several collateral initiatives related to sepsis management and antibiotic prescribing.

Nevertheless, some ED professionals reported that, within the framework of a very busy workflow and workload, there were team communication issues in the department, which meant that the sepsis digital alert could not always be acknowledged and acted upon timely

The pandemic and staff changes in the outreach team for sepsis meant that they felt that their role was not always known in ED; this might also be because at the time of this study, the 2 nurses in this team had been in their role for 8 or 9 months only. The team for sepsis was infrequently in person in the ED or onward, reporting occasional feelings of being negatively perceived as wanting to interfere with the work of colleagues. Some site 2 ED participants wished they had a 24/7 outreach team for more support with emergencies to alleviate workload which was perceived as untenable. The planned expansion of the outreach team for sepsis, with more staff and aiming to be operative 24/7, is a promising response on behalf of this trust to the heavy workload of professionals in ED and trust-wise.

Site 3 presents yet another different configuration of elements. Over the decade of DS use, lessons have been learned about the counterproductive effects of having too many soft-stop and pop-up alerts, and these have been actively reduced. The rollout of the sepsis digital alert was accompanied by, and optimized via, weekly flat-hierarchy, multidisciplinary meetings on sepsis awareness and optimized based on how the sepsis digital alert should work and look like. Site-3 participants involved in those meetings found them informative and useful. This initiative, together with the trust’s ongoing investment in sepsis education and training in the DS and the sepsis digital alert, appeared to support better use of the digital tool. This was the case even though the sepsis digital alert being nested within the same DS as site 2 and also described as not user-friendly. In site 3, however, the sepsis digital alert dialogue box is different, with tailored interface to the HCP role (whether a prescriber or a nonprescriber is logged-in). An aspect that was reported as needing improvement was the Situation-Background-Assessment-Recommendation form, which was often done ex post as paperwork, and therefore, did not fulfill its full potential in speeding up referrals. Finally, the support of a nurse-led, 24/7 outreach team was an extra resource for staff and the NEWS2 score was fruitfully used in combination with the sepsis digital alert.

**Table 2:** Main characteristics of National Health Service trusts’ research sites.

Characteristics			Site 1		Site 2		Site 3
**The EHRs^a^ DS^b^**							
	When DS introduced	DS was introduced in 2019		DS was introduced in 2015		DS (same as site 2) was progressively introduced from 2013	
	DS interface and access	DS interface user-friendly and the same across the site and job roles		DS interface not straightforward, with numerous tabs and colours; DS is different in intensive care		DS interface not straightforward, with numerous tabs and colours; different DS login for nurses and doctors	
	Digital alerts in the DS	Other alerts in use, mainly passive ones (eg, for ED^c^ triaging)		Other soft-stop digital alerts, as well as passive ones (eg, for ED triaging)		Other soft-stop digital alerts, as well as passive ones (eg, for ED triaging)	
	Additional functions of the DS	DS has several functions, used as a phone app and linked to tablets for patients’ observations		Use of DS’s additional functions was not found		Other software and applications are in use, eg, on sepsis management and antibiotics administration	
**The sepsis or deterioration digital alert**							
	When sepsis digital alerts introduced	Alerts was introduced in 2019		Alerts was introduced in 2016		Alerts’ was introduced in phases from 2016	
	Features of the sepsis digital alert	Sepsis digital alert is NEW2^d^ (sepsis is suspected with a score of 5+ and a confirmed or suspected infection)		Sepsis digital alert is based on in-house adaptation of sepsis red flag screening tool		Sepsis digital alert is built in the DS, based on St John Sepsis Algorithm; alert informed by a binary alarm: (1) for potential sepsis and (2) for potential severe sepsis	
	Sepsis digital alert’s functioning	Passive icon that changes color according to the score		Alert is both soft-stop and passive; prescribers and nonprescribers can respond differently		Alert is both soft-stop and passive; prescribers and nonprescribers can respond differently	
	Sepsis digital alert’s interface	On the wards, alert embedded in EHR and does not pop up; in ED, alert appears on the digital list of patients		On the wards, the alert box pops up when an EHR is opened and closed; in ED, the alert is also a passive, colorful icon on patients’ digital dashboard		On the wards, the alert box pops up when an EHR is opened and closed; in ED, the alert is also a passive, colorful icon on patients’ digital dashboard	
**Trust actions on sepsis, the DS and the sepsis digital alerts**							
	Trust’s position	Deterioration and acuity are the priority		Sepsis was among the trust’s priorities up until COVID-19; there were several initiatives for sepsis awareness raising and education (eg, sites’ performance competitions, videos and information on the intranet, sepsis trolleys, and champions)		Sepsis was among the trust’s priorities up until COVID-19; there were several initiatives for sepsis awareness raising and education (eg, sites’ performance competitions, videos and information on the intranet, sepsis trolleys, and champions)	
	Sepsis digital alerts’ roll out	ED led on alerts’ implementation		ED led on alerts’ implementation		Weekly flat-hierarchy, multidisciplinary meetings on sepsis awareness and alert’s optimization	
	Approach to digital alerts	Keep a minimal number of soft-stop digital alerts		Digital alerts were reported as too numerous		Keep a minimal number of soft-stop digital alerts	
	Trust’s deterioration or sepsis team	NEWS2-based, nurse-led, 24/7 outreach team		Sepsis team for awareness and training, and to support hospital units following sepsis digital alerts; active during office hours.		NEWS2-based, nurse-led, 24/7 outreach team	

^a^EHR: electronic health record.

^b^DS: digital system.

^c^ED: emergency department.

^d^NEWS2: National early warning score 2.

## Discussion

### Principal Findings

Participants generally viewed sepsis digital alerts positively but emphasized that they cannot substitute the HCP’s knowledge and experience in the identification and management of sepsis. Sepsis digital alerts are only a piece of the puzzle in a patient’s presentation, as well as in the complex, multimodal, and multidisciplinary clinical practice. Participants considered them as useful, context-specific reminders prompting HCPs to take a range of actions, from reviewing to escalating a patient.

Participants identified features of better sepsis digital alerts, such as accessibility and user-friendliness. More sophisticated sepsis digital alerts should be more specific; be patient based; target HCP teams; be portable and remotely accessible; and integrate community, ambulance, and primary care with secondary care to accelerate ED triaging.

Factors pertaining to the individual HCP, the hospital unit, the trust, and the digital tool itself differently combine and were seen to affect the use of sepsis digital alerts in the 3 trusts in this study. The combination of these 4 sets of factors leading to the more optimal use of a sepsis digital alert include a general, non-ICU with a lower senior decision maker:patient ratio; HCP’s previous experience with the sepsis digital alert; presence of a 24/7 emergency outreach team; sepsis digital alert’s quality improvement initiatives and continuous sepsis training; strong technological resources in the trust; good staffing and teamwork; digital tool’s ease-of-use; and digital tools that are not numerous and intrusive.

### Strengths and Limitations

We included 3 NHS hospital trusts in this study to explore differences in relation to the EHRs DS and the sepsis digital alert, the implementation optimization strategies, and the approach and training in relation to sepsis. The study set out to involve ED, outreach or sepsis teams, and ICU professionals in different roles and career stage; as a result, we obtained a varied sample. The multisite design and the varied professional profiles afforded meaningful comparisons across job roles, units, and trusts toward a more nuanced identification of factors affecting the use of the sepsis digital alerts. Observations provided insights on how the sepsis digital alert fitted with workflows that were richer than the self-reported descriptions of sepsis digital alert in the interviews. Finally, 2 researchers (RL and AJ Borek) with different disciplinary backgrounds conducted interviews and observations in 1 of the sites. This added rigor to the process of data collection and analysis.

All 3 sites in this study are large, high-resourced, and urban university hospitals; research in trusts with different characteristics and contexts (eg, smaller district hospitals and hospitals in lower-resources settings) could convey different results. HCPs from other hospital wards and units may have different experiences and provide a contrasting example to further understand how sepsis digital alerts are viewed and used but was beyond the resources available for this study. Due to research team capacity and time constraints dictated by the project timeline, we could not include all the 3 trusts for the observations. Finally, the study recruitment strategy resulted in sampling and nonresponse biases which contributed to the fact that certain professional categories, such as junior doctors, were absent. Although we made efforts to recruit junior doctors, none volunteered to participate. This was influenced by the high workload and limited time and capacity as the study was conducted at the time of junior doctor strikes. The absence of junior doctors is a remarkable limitation as sepsis digital alerts, as this study has also found, may be more useful for less senior health professionals.

### Comparison With Prior Work

The introduction of sepsis digital alerts rests on the evidence of the challenges to optimally identify and manage sepsis in hospitals, in particular in EDs, where HCPs have been found to need more confidence and time to assess and escalate septic patients [18,21]. Our study found that sepsis digital alerts support HCPs in identifying and making quicker decisions about deteriorating patients, which might be particularly important and helpful to new and less experienced HCPs in ED and in the general wards. The sepsis digital alert is an additional element that HCPs factor in their practice; it is not a substitute for their judgment. Similarly, a US study found that participants were more inclined to accept the machine learning–based system for sepsis if they perceived the tool as a partner, supporting their autonomy and workflow, and not a surrogate of their clinical judgment [54]. For this same reason, another work involving hospital leaders found that participant tended to distrust more machine learning than rule-based sepsis CCDSSs [55]. The literature on CCDSS implementation supports this finding: a study across 4 Italian hospitals concluded that the perception that an advanced CCDSS could reduce HCPs autonomy was the most significant barrier to implementation [56].

Users’ attitudes and perceptions about the ease of use and usefulness have been at the center of established technology acceptance theories [57,58]. Trust in the sepsis digital alert and its uptake were found to be affected by individual factors, such as previous experience with the DS, as 2 recent systematic reviews on the implementation of a CCDSS [59] and of an EHRs DS have corroborated [60]. Although our participants did not directly discuss trust in the sepsis digital alert, its importance can be inferred from other aspects they raised, especially when thinking about better sepsis digital alerts. Participants would welcome more sophisticated and reliable sepsis digital alerts, which would be more accurate and transparent, as previous work observed [61,62]. At the same time, our study revealed that perceived usefulness in the sepsis digital alert depended on the HCPs’ professional experience and specialty training. More senior and emergency HCPs as well as intensivists tended to take less advantage from the sepsis digital alert. Similarly, previous work has demonstrated that HCPs can disregard the evidence underpinning CCDSSs for fear that their critical reasoning and, again, their professional autonomy are challenged [56]; but also, because the evidence embedded in the digital tool may be seen as jeopardizing hierarchical, power relations based on medical specialty and seniority [63].

Concomitantly, features and functionalities of the digital tool appear to influence the use of the sepsis digital alert [45,46]. A study highlighted that easy-to-use tablet applications as part of the DS for sepsis were important mediators facilitating implementation [62]. We also found that accessible, mobile phone apps and functions, such as the chat of the EHRs DS in site 1, appear to support better use of the sepsis digital alert. The usability of the digital tool has been a focal aspect in theories of ergonomics and human-technology interaction [64,65]. Work on the uptake of CCDSSs found that scarcity of available computers, unfriendly user interface, and excessive number of intrusive alerts lead to disengagement and fatigue [31,56,64,66]. Our study corroborates the importance of factors inherent to the design of the sepsis digital alert and the EHRs DS; both sites 1 and 3 made the deliberate choice of keeping minimal or reducing soft-stop digital alerts.

Previous work has shown the importance of functional teamworking; this should be based on high standards of coordination and communication to ensure the smooth journey of the septic patient and improve clinical outcomes [20,61]. Our results indicate the sepsis digital alert contributes to prompter patients’ referral, escalation, and treatment. However, participants felt that lack in communication among staff could hamper the proper use of the sepsis digital alert, as some participants in site 2 raised. This resonates with the findings of the aforementioned study in the pediatric ED where professionals’ acknowledgment of the sepsis digital alert was based on factors specific to the ED environment [46]. Significantly, another study found that a discontinuous flow of communication and teamwork among clinicians was a barrier to the integration of a machine learning sepsis early warning system in ED [62].

Organizational factors were identified as a significant obstacle to recognizing and responding to patients with sepsis in ED [21]. Significantly, our study corroborated how trust-level factors, such as good level of staffing, staff training and involvement in the sepsis digital alert’ optimization, and appropriate technological resources, linked with more optimal use of these CCDSSs. In line with our results, other work concluded that organizational factors affected HCPs’ trust in the sepsis digital alert; this connection was facilitated by engagement and education activities fostering sepsis digital alerts’ understanding and acceptance [45,46,55,62]. The importance of the context in affecting individual HCPs’ decisions and practices related to CCDSSs has been demonstrated in studies using the normalization process theory [67-69]. Accordingly, organizational and practice theories applied to the introduction of technology in the complex health care environment maintain that implementation processes are connected with the interaction between the technology, on the one hand, and HCPs’ practices, teamwork relationships, organization’s policies and priorities, on the other hand [70-72]. The uniqueness of the hospital context makes it difficult to compare the effectiveness of a sepsis digital alerts in isolation.

This study highlights that the introduction of sepsis digital alerts necessitates a multilevel approach [56,72] that includes understanding and actions at 4 sets of factors: the HCP, the hospital unit or department, the hospital, and the digital tool. Multilevel approaches to innovations, including digital ones as in this study, have been captured by several process frameworks developed in implementation science. Comprehensive innovation process frameworks have provided research logic models factoring in several determinants which we found in our work. These determinants spanned from the intervention characteristics, the inner and outer settings—such as leadership, networks, and communication—and learning climate, to the characteristics of individuals and the process [73-75]. Other tools also embedded a plethora of factors to be considered when assessing intervention scalability [76]; factors worth analyzing to assess scaling readiness include the strategic or political context, the intervention costs and benefits, delivery setting, and workforce [76]. Other work has concentrated on specific factors of the scale and spread journey, such as the types of innovations [77], specific context and processes, such as the NHS innovation pathways and its accelerators [78]; the importance of the context [79]; of patient and public involvement [80]; and of innovation intermediaries [81]. The necessity of adopting a multipronged, evolving strategy which goes beyond mechanistic logics of change have been promoted [82,83]. This same approach has emerged as mandatory from our retrospective, descriptive qualitative study based on the views and uses of sepsis digital alerts already in place in 3 hospital trusts in England.

### Implications

An a priori analysis of the organizational environment to assess the hospital readiness and unique feasibility for the introduction of the sepsis digital alert is recommended. Mapping areas demanding change in the trust and planning resources and actions for their improvement mitigate the risk that sepsis or deterioration CCDSSs fail to offer the intended benefits [72]. In addition to more structural factors, such as resources, staffing, sepsis training, and a successful leadership-teamwork dynamic, it is also advisable that organizational cultural factors are factored in. Cultural factors that trusts should consider assessing include the trust’s prioritization of sepsis or of deterioration and acuity, staff retention trends and sociodemographic profile, attitudes and readiness toward technological innovations, and the more impalpable norms regulating hierarchies and power among staff.

Hospital trusts should aim to plan ongoing implementation optimization initiatives in the pilot and rollout phases [55]. These initiatives should be flat-hierarchical and multidisciplinary so that HCPs with different job roles and training, based in different hospital units, can voice their unique perspective and support needs they expect to be met by the sepsis digital alert [84,85]. The trust should ensure the continuous involvement of information technology developers [86], and that staff feedback is appropriately collected and analyzed so that it materializes into context-based modifications of the tool. The engagement of staff should go hand-in-hand with education and training to aim at the maximization of behavior changes toward improved patient care. These activities should be reinforced by the establishment of champions and other strategic communication and educational campaigns, whereby staff can easily and remotely access information about the sepsis digital alert. It would be useful if trusts established dedicated advisory groups monitoring and managing all the actions necessary for a more successful post go-live [86].

Design, content, and technical aspects can act as barriers or facilitators to changing the clinical behavior toward better patient care in technology-based interventions [87]. Further multidisciplinary research should inform the development of sepsis digital alerts which are easy to use but at the same time are more sophisticated, able to target specific HCPs and hospital units, and simultaneously become more patient-specific, transparent, and interactive [88]. Sepsis digital alerts should also be more effectively linked to guidance on sepsis protocols, escalation practice, and antibiotics prescribing. Researchers and developers should work in conjunction with HCPs and policy makers to refine technology-based behavior change techniques that *effectively* support HCPs’ decision-making, care practice, and improve patients’ outcomes as a result.

### Conclusions

Current sepsis digital alerts nested within the EHRs are introduced to support the identification and management of sepsis or deterioration and improve patient outcomes. These sepsis or deterioration CCDSSs fulfill their purpose but not entirely and not equally in all hospitals; an organic, multilevel framework to enhance tailored implementation of sepsis digital alerts is needed, along with the simultaneous validation of their effect on patient outcomes. No technological innovation in the health care setting can be a solo driver of change, and sepsis digital alerts are not magic wands able to dissipate issues that instead become important barriers to their optimal use in the rollout phase. Sepsis digital alerts implemented in trusts with good levels of staffing, resources, and functional teamwork are likely to be taken up more optimally and become good partners for HCPs. Equally, where the roll out of sepsis digital alerts is accompanied by multidisciplinary quality optimization initiatives, and by training, education, and other sepsis awareness actions that continually engage staff, HCPs are more likely to accept and embed the sepsis digital alert in their practice. Trust implementation of sepsis digital alert requires changes on multiple levels and at all phases of the intervention, starting from a prego-live analysis assessing and addressing organizational needs and readiness. Advancements toward minimally disruptive and smart sepsis digital alerts, which are more accurate and specific, but at the same time scalable and accessible, have to see policy changes and investments in multidisciplinary research agendas.

## Appendix

Multimedia Appendix 1 Interview topic guide.

## Abbreviations

CCDSS: computerized clinical decision support system
DiAlS: Digital Alerting for Sepsis
DS: digital system
ED: emergency department
EHR: electronic health record
HCP: health care professional
ICU: intensive care unit
NEWS: national early warning score
NHS: National Health System
