# Supplementary material for: Views and Uses of Sepsis Digital Alerts in National Health Service Trusts in England: Qualitative Study With Health Care Professionals
Source: JMIR Hum Factors. 2024 Oct 15;11:e56949. doi: 10.2196/56949 (PMC11522658; doi:10.2196/56949)
Supplement: Multimedia Appendix 1 [file humanfactors_v11i1e56949_app1.docx]

# DiAlS Qual

**Healthcare Professional Topic Guide**

## Briefing

1. Welcome and thanks to participant for agreeing to take part.
2. Introduce self.
3. The aims of this study are described in the participant information sheet. We are interested in your views and experiences, there are no right or wrong answers. For example, if you haven’t used any alerts or tools to manage patients with sepsis we would still like to hear your views. Differences between professionals may arise from different perceptions, priorities, and experiences. These differences are important to us and we value your unique perspective.
4. If at any time during the interview you do not wish to answer a question, that’s okay.
5. I would like to audio record our conversation. The recording will be transcribed, but everything you say will be anonymous. Your name and any names you mention, and any places you mention will be taken out, so that if someone read your interview transcript, they would not know who you are or where you work.
6. Your interview will remain confidential.
7. If, at any stage, you wish to stop the audio recording, please let me know.
8. Do you have any questions?

## Topics to be explored

Below is a list of topics to be discussed. The topic guide will remain flexible with respect to what is of importance to participants.

1. Participant’s experience of working in hospital and managing patients with (suspected) sepsis.
2. Participant’s experience of using any alert, algorithm or protocol to help identify patients with (suspected) sepsis.
3. Participant’s experience of developing and/or implementing digital alerts to identify patients with sepsis (where applicable).
4. Participants experience of introducing protocols or alerts to identify patients who are deteriorating/with suspected sepsis.

**Example questions with prompts** (additional questions may be added during interviews following the topics above):

1. DEFINITION - First of all can you tell me, in your own words, what sepsis is?
   1. How would you explain sepsis to a lay person?
   2. What is your definition of sepsis?
   3. Is there consensus in your Trust on what sepsis is/described?
   4. Is sepsis currently a major priority or not in your hospital? Was it more so before?
2. LANGUAGE with PATIENTS- How do you explain a sepsis diagnosis to a patient?
   1. What do you say to patients when you suspect they may have sepsis?
   2. How do you discuss sepsis once diagnosed is confirmed?
   3. How do you explain treatment to patients?
   4. What do you say to patients about recovery from sepsis?
   5. How is communication different with patients who already had sepsis in the past (if at all)?
3. TREATMENT/MANAGEMENT PROTOCOL - How are patients with suspected sepsis and with sepsis treated?
   1. Who is responsible for treating patients with sepsis? In A&E? On the ward?
   2. How are patients with sepsis monitored?
   3. How has your management of patients with sepsis changed over time (if at all)?
   4. How are management and treatment different with patients who already had sepsis in the past (if at all)?
4. TREATMENT/MANAGEMENT EXPERIENCE - Could you tell me about your experience of treating patients with sepsis or deteriorating patients?
   1. How commonly do you see patients with sepsis?
   2. How commonly do you see patients who have had more sepsis episodes?
   3. How often is suspected sepsis flagged?
   4. What are the main barriers and facilitators in sepsis early identification and management (pre-DAs)?
5. DIGITAL ALERTS FUNCTIONING - **How do digital alerts for sepsis fit into workflows?**
   1. When do alerts appear when someone is using the EHR?
   2. Where are alerts seen on the system?
   3. How easy is it for clinicians to acknowledge this alert and take action accordingly (ie, alert links to treatment (including antibiotic prescriptions) or clinical tests/cultures?
   4. What are the possible scenarios at the firing of a digital alerts?
   5. How would a better/ideal DSA look like? [Would it trigger a two-stage process (DA 1: alert on those potentially eligible for screening / DA 2: alert to screen for sepsis tout court)? Would you include the possibility of viewing or inserting more information re the patients (eg, previous diagnosis of sepsis or other, procedure, lab results – these not available in ED)? Could alerts appear at a better time/in a better location?]
   6. Do you have other DAs firing? How do you see the management of multiple DAs and is the one for sepsis any different?
6. DIGITAL ALERTS IMPACT- **How do digital alerts impact the decision-making of clinicians, and the treatment and management of (suspected) septic patients?**
   1. What are the differences across units/departments in relation to firing alert (if at all)? For example, what impact do A&E waiting times have on sepsis alerts firing and being responded to/acted on?
   2. What are the differences across shifts, time of the day and seasons in relation to firing alert (if at all)? For example, during flu season do they fire more often?
   3. Are you aware of any non-clinical factors affecting the use of DAs (e.g., trust in the alerts/technology, experience of the HCP, HCP position in the Trust)?
   4. Have DAs impacted on the way sepsis is conceived, by contributing to the establishment of greater consensus?
   5. Have DAs impacted the communication, patient’s handovers or other aspect of the workflow and patient flow, both within the unit team as well as that across specialty teams in the Trust and how?
   6. If the patient had sepsis already, does this change the approach to the digital alert?
   7. Are patient/carers aware of alerts and does this increase communications around sepsis, in your view?
   8. What works well and what could be improved in the digital alerts, their use and their fitting into the workflows and teamwork?
   9. What are the main barriers and facilitators in sepsis early identification and management via the support of DAs)?
7. **SEPSIS DIGITAL ALERTS IMPLEMENTAITON AND EVALUATION – ONLY For participants who have been involved in implementation of alerts in their trust:**
   1. What role have you had in implementing deteriorating patient/sepsis alerts in your Trust?
      1. Why were the alerts/protocols introduced and adopted?
      2. Who else was involved in implementation of the alert?
      3. How was the alert/protocol developed or trialled?
      4. How was the implementation planned (including any engagement strategy, and ongoing associated quality improvement processes as well as training)?
   2. What evaluation has been done on the alert?
      1. What feedback have you had from clinicians on using/responding to the alert?
      2. Has any evidence of alert fatigue been collected?
8. GENERAL/SUMMARY - What works well VS what could be improved about how patients with sepsis are identified and cared for in your hospital?
   1. Prompts: Digital alerts/other technologies, guidance, protocols, **training**, teamwork and communication, leadership.
9. FREE ADDITIONS - Is there anything else that you’d like to mention about identifying and caring for patients with sepsis?
10. SUGGESTIONS - Is there something else that we should be asking others, both colleagues and patients/family member in future interviews?
11. Would you be keen to be observed/shadowed in your clinical practice?
12. [if yes] Would you like to have a short discussion now on that part of the research? We will contact you soon to send you the PIS in relation to the study observations, and you will have more time to ask more questions and ponder your decision. Thank you.

**Demographic questions**

Age range, gender, ethnic background, religious orientation, qualifications, job title, years of experience in their clinical role at the time of consent
